# Hijacking the human complement inhibitor C4b-binding protein by the sporozoite stage of the *Plasmodium falciparum* parasite

**DOI:** 10.3389/fimmu.2022.1051161

**Published:** 2022-11-21

**Authors:** Ayman Khattab, Mikel Rezola, Marta Barroso, Mikael Kyrklund, Tero Pihlajamaa, Tobias L. Freitag, Geert-Jan van Gemert, Teun Bousema, Perttu Permi, Ossi Turunen, Robert Sauerwein, Adrian J. F. Luty, Seppo Meri

**Affiliations:** ^1^ Department of Bacteriology and Immunology, Haartman Institute, and Translational Immunology Research Program, University of Helsinki, Helsinki, Finland; ^2^ Department of Nucleic Acid Research, Genetic Engineering and Biotechnology Research Institute, City of Scientific Research and Technological Applications, New Borg El-Arab, Alexandria, Egypt; ^3^ Department of Bioproducts and Biosystems, Aalto University, Espoo, Finland; ^4^ Department of Clinical Chemistry, HUSLAB, Helsinki University Hospital, HUS Diagnostic Center, Helsinki, Finland; ^5^ Department of Medical Microbiology, Radboudumc, Nijmegen, Netherlands; ^6^ Department of Chemistry, Nanoscience Center, University of Jyväskylä, Jyväskylä, Finland; ^7^ Department of Biological and Environmental Science, University of Jyväskylä, Jyväskylä, Finland; ^8^ School of Forest Sciences, University of Eastern Finland, Joensuu, Finland; ^9^ TropIQ Health Sciences, Nijmegen, Netherlands; ^10^ Université de Paris, MERIT, IRD, Paris, France; ^11^ HUSLAB Diagnostic Center, Helsinki University Central Hospital, Helsinki, Finland; ^12^ Department of Biomedical Sciences, Humanitas University, Milan, Italy

**Keywords:** complement evasion, Plasmodium, sporozoites, circumsporozoite protein, C4b binding protein

## Abstract

The complement system is considered the first line of defense against pathogens. Hijacking complement regulators from blood is a common evasion tactic of pathogens to inhibit complement activation on their surfaces. Here, we report hijacking of the complement C4b-binding protein (C4bp), the regulator of the classical and lectin pathways of complement activation, by the sporozoite (SPZ) stage of the *Plasmodium falciparum* parasite. This was shown by direct binding of radiolabeled purified C4bp to live SPZs as well as by binding of C4bp from human serum to SPZs in indirect immunofluorescence assays. Using a membrane-bound peptide array, peptides from the N-terminal domain (NTD) of *P. falciparum* circumsporozoite protein (CSP) were found to bind C4bp. Soluble biotinylated peptide covering the same region on the NTD and a recombinantly expressed NTD also bound C4bp in a dose-dependent manner. NTD-binding site on C4bp was mapped to the CCP1-2 of the C4bp α-chain, a common binding site for many pathogens. Native CSP was also co-immunoprecipitated with C4bp from human serum. Preventing C4bp binding to the SPZ surface negatively affected the SPZs gliding motility in the presence of functional complement and malaria hyperimmune IgG confirming the protective role of C4bp in controlling complement activation through the classical pathway on the SPZ surface. Incorporating the CSP-C4bp binding region into a CSP-based vaccine formulation could induce vaccine-mediated immunity that neutralizes this immune evasion region and increases the vaccine efficacy.

## Introduction

The complement system is an important component of both human innate and adaptive immune defense (1). It opsonizes microbes for recognition by phagocytic cells directly or after activation by antibodies. It also forms membrane attack complexes (MACs) on the microbial surface membranes to induce cell lysis. Complement is also a key system in clearing immune complexes and apoptotic cells from the circulation and in delivering antigens for the induction of specific immune responses.

Because of the multiple roles of complement and its potential to cause cell damage, its activation needs to be tightly regulated. A set of soluble and membrane bound complement inhibitors exists to control complement activity and prevent damage to host cells (2). Pathogens may mimic host cells by exploiting the soluble regulatory proteins to escape complement attack (3, 4). Factor H (FH) and the C4b binding protein (C4bp), the soluble regulators of the alternative (AP) and classical/lectin (CP/LP) pathways of complement activation, respectively, are the most exploited regulators by pathogens. FH and C4bp are hijacked from the circulation by specific surface proteins of the pathogens to inactivate complement components on the surfaces.

Although several complement evasion mechanisms and molecules have been identified for many pathogens during the past decades, it was only relatively recently that evasion mechanisms and molecules have been identified for the *Plasmodium falciparum* parasite. Simon et al, 2013 (5) reported the acquisition of FH from human blood by the GAP50 protein on the surface of the extracellular *P. falciparum* gametes in the mosquito’s midgut. In another attempt to investigate complement evasion by the developmental stage of the malaria parasite, Kennedy et al, 2016 (6) demonstrated recruitment of FH to the merozoite surface by the merozoite surface protein Pf92. In a more recent work, Kennedy et al, 2017 (7) additionally showed that *P. falciparum* merozoites can also inhibit the classical pathway of complement activation by recruiting the soluble regulator C1 esterase inhibitor (C1-INH) that negatively regulates the classical and lectin pathways. However, this is an uncommon mechanism compared to exploiting C4bp by microbes. PfMSP3.1 was identified as the C1-INH interacting partner (7). Merozoites have also been reported to bind C1-INH to their surfaces through an interaction with glycan moieties within the *P. falciparum* glycosylphosphatidylinositol (*Pf*GPI) molecule (8).

The sporozoite (SPZ) is another developmental stage of the *Plasmodium* parasite that exists extracellularly and is exposed to the complement system throughout its journey from the skin to the liver. So far, no complement evasion mechanisms have been discovered for this invasive stage. Nevertheless, malaria exposed populations as well as chemoprophylaxis-treated and SPZ immunized volunteers were shown to carry antibodies that can fix and activate complement (complement fixing antibodies) on the SPZ surface through the classical pathway of complement activation, interfere with SPZ membrane integrity and inhibit SPZ infectivity *in vitro* (9). High levels of the anti-SPZ complement fixing antibodies were also shown to associate with protection against clinical malaria (10). Therefore, enhancing complement activation on the SPZ surface by neutralizing potential complement evasion mechanisms would elicit stronger pre-erythrocytic immunity against malaria. C4bp is the primary fluid-phase inhibitor of the C3 convertase of CP/LP. It has a decay-accelerating activity for the classical pathway C3 convertase and acts as a cofactor promoting factor I-mediated cleavage of C4b. C4bp is a 570 kDa multimer present in human plasma (250µg/ml) in several oligomeric forms (11). The main form of C4bp is composed of seven identical α-chains and one β-chain. Other forms of C4bp contain, six α-chains and one β-chain, 7 α-chains and no β-chain, or 6 α-chains and no β-chain. The C4bp α- and β-chains are attached by disulfide bonds at their C-terminal and radiates from a central core giving C4bp a spider- or octopus-like structure (12). Each α-chain (75 kDa) comprises eight complement control domain proteins (CCPs) and a C-terminal oligomerization domain. The β-chain (40 kDa) of C4bp has only three CCPs and a C-terminal oligomerization domain. The β-chain shares no sequence homology with the α-chain. Most pathogens capture C4bp *via* its CCP1-2 domains of the α-chains (13) resulting in protection from complement mediated lysis.

In this report, we show that SPZs capture C4bp to their surfaces *via* the SPZ major surface circumsporozoite protein (CSP). We mapped the C4bp binding site on CSP to its N-terminal domain (NTD). This interaction was shown to involve the CCP1-2 domains of the C4bp α-chain. Preventing C4bp binding to the SPZ surface negatively affected the gliding motility of the SPZs in the presence of functional complement and malaria hyperimmune IgG. Incorporating the CSP functional region (the C4bp- binding motif) into a CSP-based vaccine formulation could induce vaccine-mediated immunity that neutralizes this immune evasion region and increases the vaccine efficacy.

## Results

### Binding of radiolabeled C4bp to the SPZ surface

**Figure 1:**
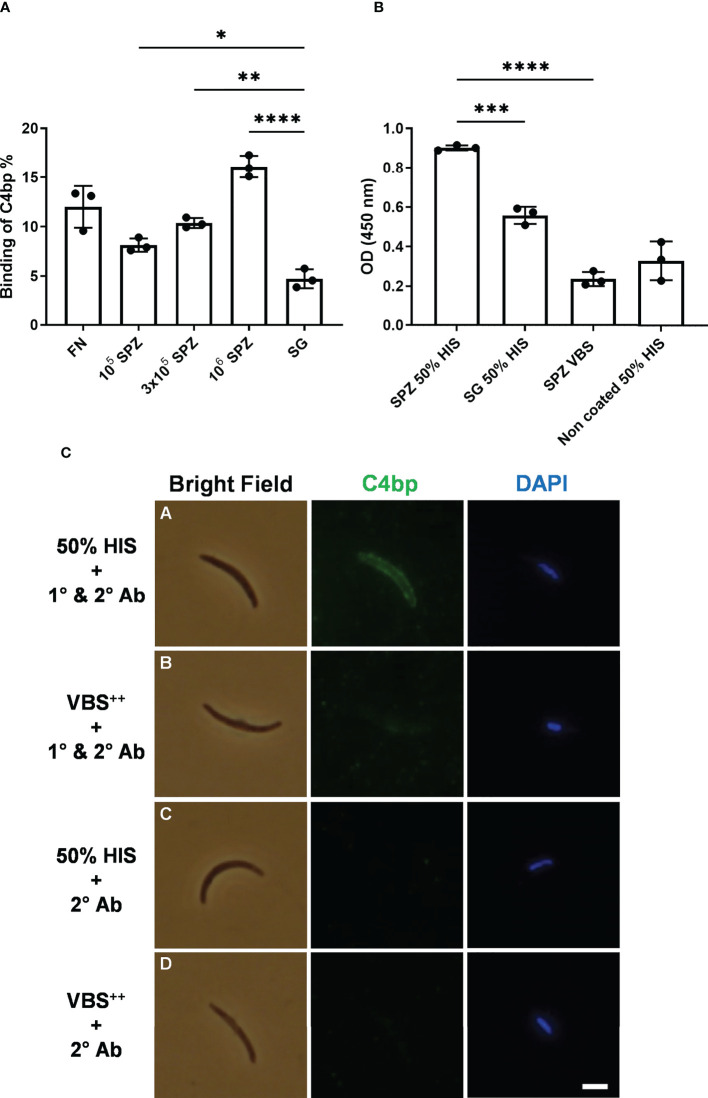
Binding of C4bp to SPZs. **(A)** Radiolabeled C4bp was incubated with *Fusobacterium necrophorum* (FN) bacteria (positive control) and with different amounts of *P. falciparum* SPZs or insoluble fraction of mosquito salivary glands (SG) (negative control). **(B)** Binding of serum C4bp to SPZs fixed to the wells of microtiter plates was tested in a whole-SPZ ELISA. Bound C4bp was detected using sheep polyclonal anti-human C4bp, donkey anti-sheep IgG/HRP. Comparisons between groups were analyzed by ANOVA followed by *post-hoc* Tukey’s multiple pairwise comparisons test. The asterisks represent a significant difference between groups. **(C)** Binding of serum C4bp to the SPZ surface in indirect immunofluorescence assays (IFA). SPZs were incubated with either HIS **(A, C)** or VBS++ **(B, D)** and subjected to IFA. **(A, B)** The α-chain of C4bp (C4bp-α) was labeled on the SPZ surface using primary antibodies (1°) against C4bp-α and detected with Alexa Fluor488-labeled (green) secondary antibody (2°). **(C, D)** As a secondary antibody control, SPZs were incubated with secondary antibody in the absence of primary antibody. Nuclei were detected by DAPI staining (blue). Asterisks indicate the p values: p < 0.05 = *; p < 0.01 = **; p < 0.001 = ***, and p < 0.0001 = ****. Scale bar, 3 µm.

SPZs freshly isolated from salivary glands of *P. falciparum*-infected mosquitoes were tested for their ability to bind radiolabeled C4bp (^125^I-C4bp) in 20% sucrose tube assays. Three SPZ counts, 10^5^, 3x10^5^ and 10^6^ SPZs, were used in each assay in addition to 4×10^7^ cells/assay of the bacterium *Fusobacterium necrophorum* as a positive control for C4bp binding to cell surface (14). Salivary glands’ insoluble fraction (equivalent to those that yielded 10^6^ SPZs) of uninfected mosquitoes were also used as a negative control for C4bp binding. C4bp was found to bind to *P. falciparum* SPZs in a dose-dependent manner (**Figure 1A**). Maximum reported binding percentage (relative to total radioactivity in the assay tube) was 16.2% with 10^6^ SPZs that was significantly different from C4bp binding the salivary gland’s insoluble fraction (negative control) assay condition (4.8%). Binding percentage of C4bp to 3x10^5^ SPZs (10.4%) and 10^5^ SPZs (8.3%) were also significant compared to the negative control. C4bp binding was also confirmed to the positive control cells (11.5% binding).

### Binding of serum C4bp to the SPZ surface in ELISA and indirect immunofluorescence assays

To confirm C4bp binding to the *P. falciparum* SPZs that was observed in the direct binding assays, we tested the ability of SPZs to recruit C4bp from human serum using whole-SPZ ELISA and indirect immunofluorescence assays (IFA). In whole-SPZ ELISA, 10^4^ SPZs/well or salivary glands’ insoluble fraction (equivalent to those that yielded 10^4^ SPZs) from uninfected mosquitoes were fixed to the wells of microtiter plates and overlayed with either 50% HIS (heat inactivated serum) in VBS^++^ (1x veronal buffered saline, pH 7.3, 0.15mM CaCl_2_, 0.5mM MgCl_2_) or VBS^++^ alone. After detecting C4bp binding with sheep anti-C4bp antibodies it was observed that SPZ bound significantly more C4bp from human serum than did the salivary gland debris control, thus, supporting the interaction between *P. falciparum* SPZs and human C4bp. In indirect immunofluorescence assays, SPZs were incubated with either 50% HIS or VBS^++^ for 30 minutes followed by extensive washing to remove non-specifically bound proteins. C4bp binding to the SPZ surfaces was detected with sheep anti-C4bp followed by anti-sheep-AF488 secondary antibody. The fluorescence staining revealed that the SPZ surface was positive for bound C4bp when treated with 50% HIS (**Figure 1C**, panel A). However, the binding signals for C4bp were observed to vary from strong to weak, i.e. not all SPZ showed strong binding of C4bp to their surfaces. This finding was investigated further (please, see a later section of the results). Controls with VBS^++^ instead of HIS remained negative indicating lack of nonspecific binding of the antibodies used (**Figure 1B**, panel B). No C4bp binding signals were detected in the 50% HIS incubated SPZs in the absence of primary antibody (**Figure 1B**, panel C) indicating specificity of anti-C4bp staining. These results demonstrated that C4bp was recruited to the *P. falciparum* SPZs surface from human serum.

### Binding of C4bp to CSP peptides immobilized to cellulose membrane

**Figure 2:**
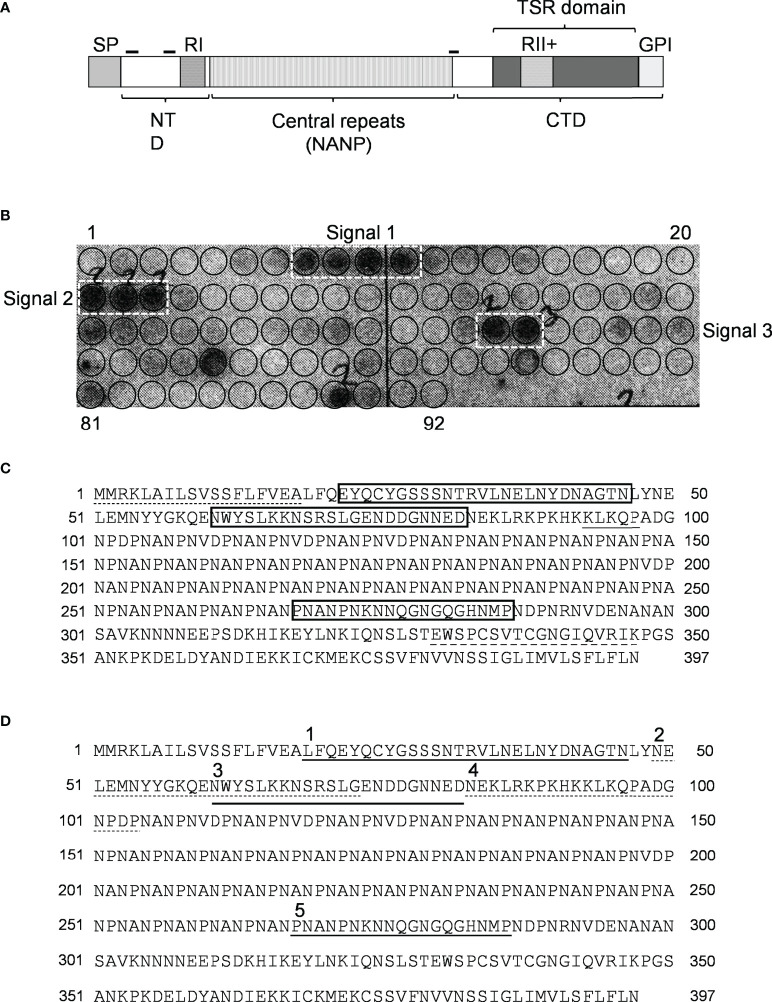
Binding of C4bp to CSP peptides. **(A)** Schematic representation of *P. falciparum* CSP. SP, signal peptide; NTD, N-terminal domain; CTD, C-terminal domain; RI, region I; RII+, region II plus; GPI, GPI anchor sequence; hyphen symbols denote locations of C4bp binding signals. **(B)** PepSpot membrane showing C4bp binding signals to CSP peptides. Circles drawn on membrane indicates locations of the 92 overlapping peptides. **(C)** C4bp binding signals delineated by rectangles on the CSP amino acid sequence. Dash, single, and dash long line under specific amino acid sequences indicates SP, RI and RII+ regions, respectively. **(D)** Synthesized biotinylated CSP peptides delineated on the FL-CSP polypeptide sequence. Number 1, 3, and 5 above the sequence denote Pep1, 3, and 5 that showed binding to C4bp in the PepSpot assay, respectively, and number 2 and 4 denote the control peptides Pep2 and 4, respectively.

Since CSP (**Figure 2A**) is the major SPZ surface protein and the main target of the host’s immune response against the pre-erythrocytic stage of the malaria parasite (15, 16) it was tested as a potential mediator of resistance to complement activity *via* recruiting C4bp to the SPZ surface. Peptides spanning the full-length CSP (FL-CSP) sequence, residues 1–397 (**Figure 2C**), were synthesized as 92 16-amino acid-long peptide spots (PepSpot) (17) with a three amino acid shift from one spot to the next (13 amino acid overlap) and covalently attached *via* carboxyl termini to polyethylene glycol-derivatized cellulose. In a peptide scanning assay using the CSP PepSpot membrane, C4bp was found to bind to distinct peptides on the array. Three groups of signals (signals from 2 or more adjacent peptides) for binding of C4bp to CSP peptides were detected (**Figure 2B**. dotted white boxes). Two of these were at the NTD of CSP upstream of the repeat region while the third was at the junction between the repeat region and the C-terminal domain (CTD).

### Binding of biotinylated CSP peptides to C4bp and CCP1-2 of the C4bp α-chain

**Figure 3:**
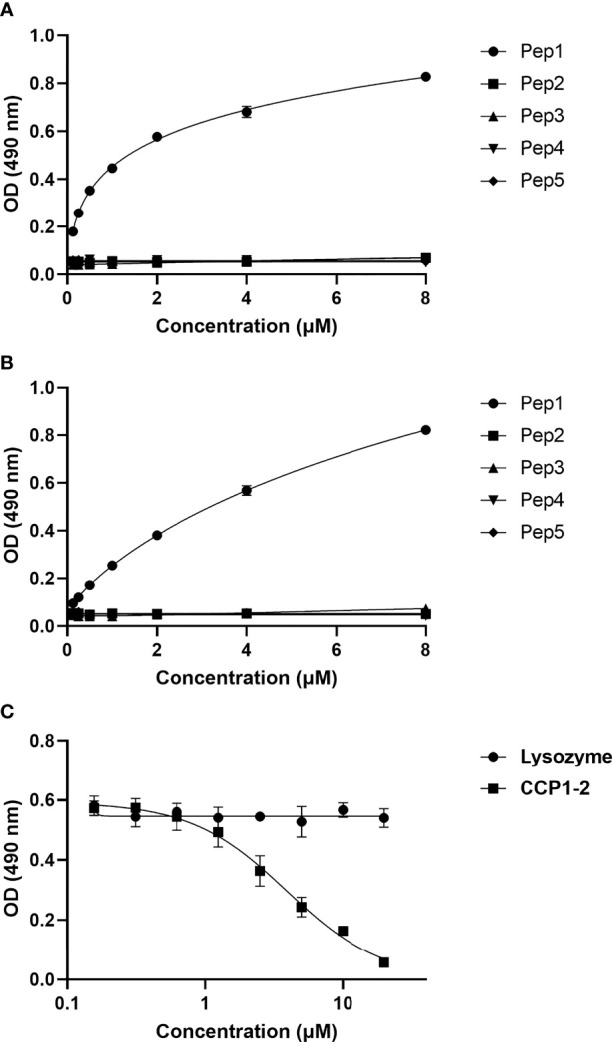
Binding of CSP biotinylated peptides to C4bp and CCP1-2 domains of the C4bp α-chain. **(A)** Binding of CSP Pep1, 3 and 5 (representing the 3 positive binding signals observed in the peptide array analysis) and Pep2 and 4 (negative control peptides) to C4bp. **(B)** Binding of the CSP peptides to CCP1-2 domains. **(C)** Inhibition of Pep1 binding to C4bp by CCP1-2 domains.

Binding of CSP peptides to C4bp was further studied by ELISA-based binding assays. Three CSP biotinylated peptides, Pep1, Pep3 and Pep5 (**Figure 2D**) representing the three PepSpot C4bp binding signals (**Figure 2B**) were synthesized. Two control peptides were also synthesized (Pep2 and Pep4). Control Pep2 (**Figure 2D**) was designed to overlap with the two lysine residues present in Pep3, while control Pep4 was designed to include Region I (RI) known to play a role in SPZ attachment to host cells (18) and to be a cleavage site for the NTD of CSP (19). In the ELISA binding assays Pep1 representing the four positive spots (signal 1) on the CSP peptide array was the only peptide that bound significantly to the immobilized C4bp (**Figure 3A**). On the other hand, Pep3 and Pep5 representing signal 2 (3 positive spots) and signal 3 (2 positive spots), respectively, bound poorly to immobilized C4bp. ELISA binding assays using immobilized CCP1-2 of the C4bp α-chain known to contain the binding site on C4bp to many pathogens (13) were also performed. Again, Pep1 bound significantly to CCP1-2 (**Figure 3B**) suggesting that indeed C4bp interact with CSP *via* its N-terminal CCP1-2 domains. Additionally, in an inhibition of binding assay, CCP1-2 inhibited binding of Pep1 to C4bp in a dose dependent manner (**Figure 3C**) confirming the specificity of Pep1 binding to CCP1-2 on the immobilized C4bp. Lysozyme was used as a control for the inhibition of binding assay.

### Binding of recombinant CSP 6-histidine-tagged NTD to C4bp in ELISA format assays

**Figure 4:**
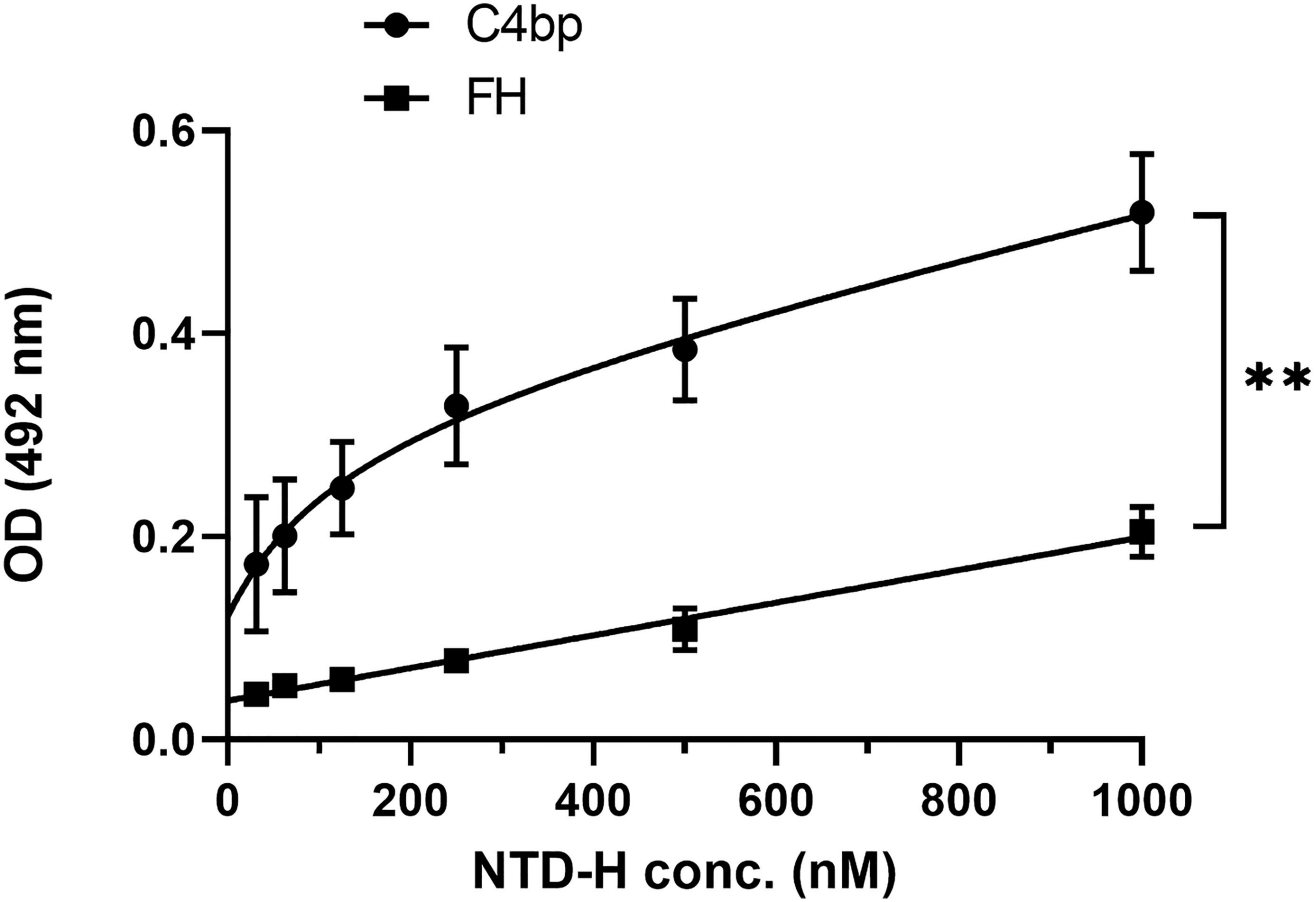
A dose response analysis of NTD-H binding to C4bp and to the alternative pathway regulator FH. The binding experiment was done by ELISA. Comparison between the two data sets was analyzed by the t test. p < 0.01 = **.

To confirm that the interaction between Pep1 on the NTD of CSP with C4bp was not a result of a conformational artefact acquired by the peptide being outside the context of the entire NTD, a recombinant NTD tagged with 6 histidine (NTD-H) was expressed in *E. coli*, and purified under native conditions. In an ELISA format binding assay, recombinant NTD-H was also found to bind to immobilized C4bp in a dose-dependent manner (**Figure 4**). The complement alternative pathway regulator FH did not show significant binding to NTD-H.

### Co-immunoprecipitation of native CSP with serum C4bp

**Figure 5:**
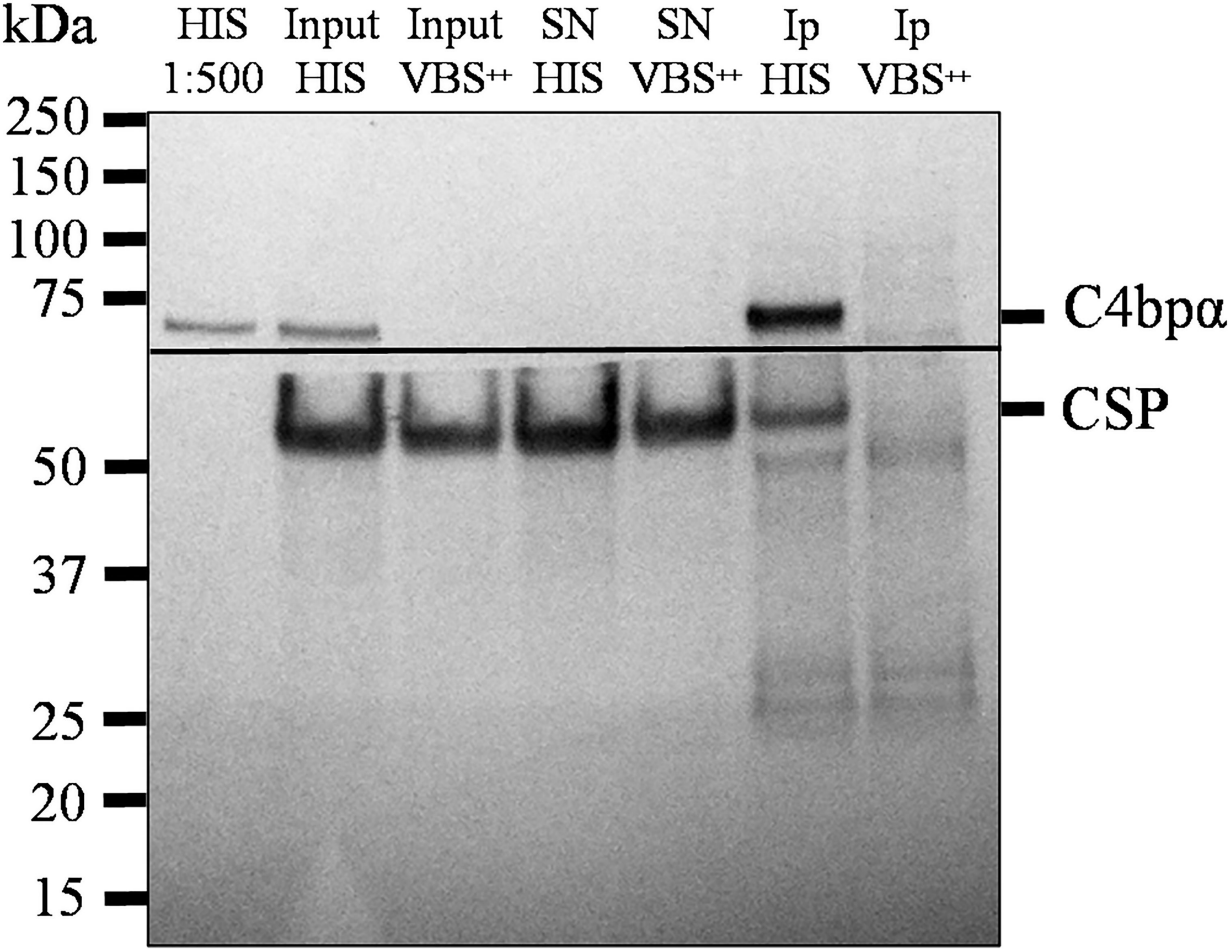
Native CSP from SPZs interacts with C4bp. SPZs were incubated with either HIS or VBS^++^. SPZs were then thoroughly washed and lysed with a lysis buffer. C4bp bound to SPZ surface proteins was immunoprecipitated from the lysate using anti-C4bp and immunoprecipitated proteins were subjected to SDS-PAGE under reducing conditions and Western blotting. The horizontal line represents the membrane separation point for the membrane pieces probed with anti-C4bp (upper piece) and anti-CSP antibodies (lower piece). HIS: heat inactivated serum; Input: starting material; SN: supernatant; IP: immunoprecipitates.

The interaction of the NTD of CSP with C4bp was also investigated using native CSP in a co-IP setup. Here, co-IP assays were performed on lysates of SPZs that were previously incubated with 50% HIS or VBS^++^and anti-C4bp. Formed complexes were precipitated by Dynabeads™ Protein G. Anti-C4bp was shown to co-immunoprecipitate native CSP from lysates of SPZs that were exposed to HIS (containing native C4bp) but not when the lysates were exposed to VBS^++^ buffer alone (**Figure 5**). This finding confirmed binding of serum C4bp to the major SPZ surface antigen CSP.

### Depletion of C4bp from serum impairs gliding motility of SPZs

Next, we wanted to test whether absence of C4bp from normal human serum (NHS) could enhance the activity of the classical complement pathway against the parasite. When SPZs are injected into the skin through a mosquito bite, they actively move by gliding motility within the dermis to find and penetrate blood vessels to enter the circulation (20–22). Impeding SPZ motility could prevent them from reaching the circulation and allow for sufficient time for the immune system to clear them while they are still within the dermis. We chose to the study the gliding motility as it is essential for SPZ infectivity and could be affected by complement activation on the SPZ surface.

We hypothesized that in the absence of C4bp, while functional complement (C4bp-depleted NHS) and a classical pathway activator (malaria hyperimmune IgG) are present, complement will get activated on the SPZ surface and the activation could have a deleterious effect on the gliding motility of the SPZs.

To test this, we incubated SPZs with purified IgG from a malaria hyperimmune serum pool in the presence of NHS (functional complement), HIS (non-functional complement), C4bp-depleted NHS (functional complement), C4bp-depleted-HIS (non-functional complement) or C4bp-depleted NHS supplemented with physiological level of C4bp for 10 minutes at 37°C. Then, SPZs were immediately subjected to gliding motility assay (23). In this assay, CSP trails are shed by the SPZs (24). The trails can be stained with an antibody against CSP and then observed and counted under an immunofluorescence microscope. The ability of complement to limit gliding motility will be inversely correlated with the number of CSP trails.

**Figure 6:**
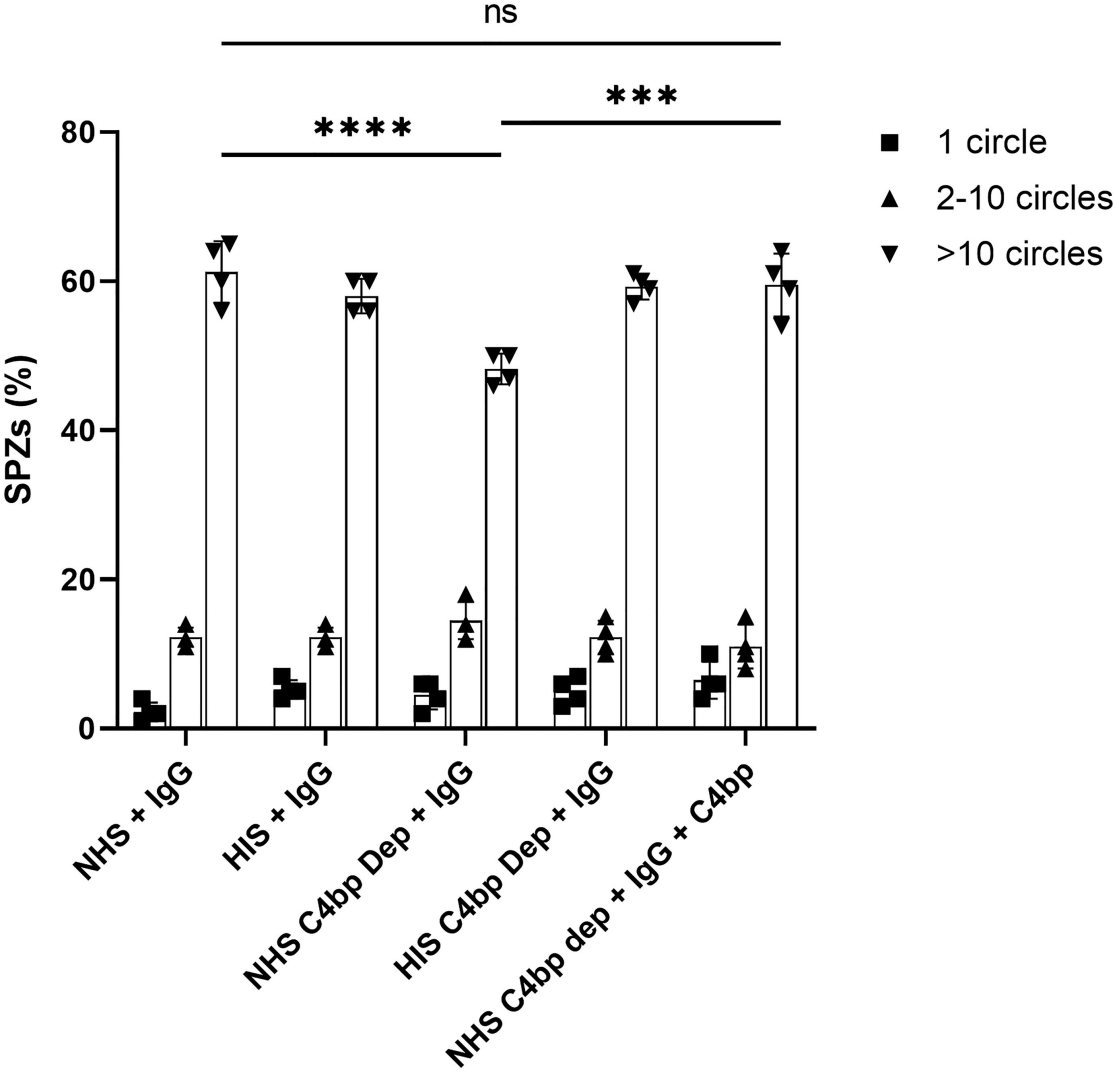
C4bp enhances SPZ resistance to malaria hyperimmune IgG -mediated complement activity. Salivary gland SPZs were pre-incubated with NHS or NHSΔC4bp (C4bp-depleted NHS), both in the presence of hyperimmune IgG, NHSΔC4bp supplemented with physiological level of C4bp in addition to hyperimmune IgG, HIS or HISΔC4bp, both in the presence of hyperimmune IgG and subjected to a gliding motility assay. The number of fluorescent circles produced by each SPZ was counted for a total of 100 SPZ per test condition and the percentages of SPZs with 1, 2-10 or >10 trail circles are shown. Comparisons between groups were analyzed by ANOVA followed by post-hoc Tukey’s multiple pairwise comparisons test. p < 0.001 = ***, p < 0.0001 = **** and not significant = ns.

The effect of the absence of the complement inhibitory activity of C4bp on the gliding motility was observed when comparing the number of >10 circles or trails among the different test conditions (**Figure 6**). Interestingly, gliding motility was significantly reduced when SPZs were exposed to hyperimmune IgG (anti-SPZ IgG) in the presence of C4bp-depleted NHS when compared to NHS (p<0.0001, **Figure 6**). The deleterious effect of the absence of C4bp was abolished when C4bp-depleted NHS was supplemented with C4bp at physiological concentration confirming the protective role of C4bp in controlling complement activation though the classical pathway on the SPZ surface. No differences between groups were observed with smaller numbers of circles or trails.

### Variable quantities of full length CSP are present on the SPZs surface

**Figure 7:**
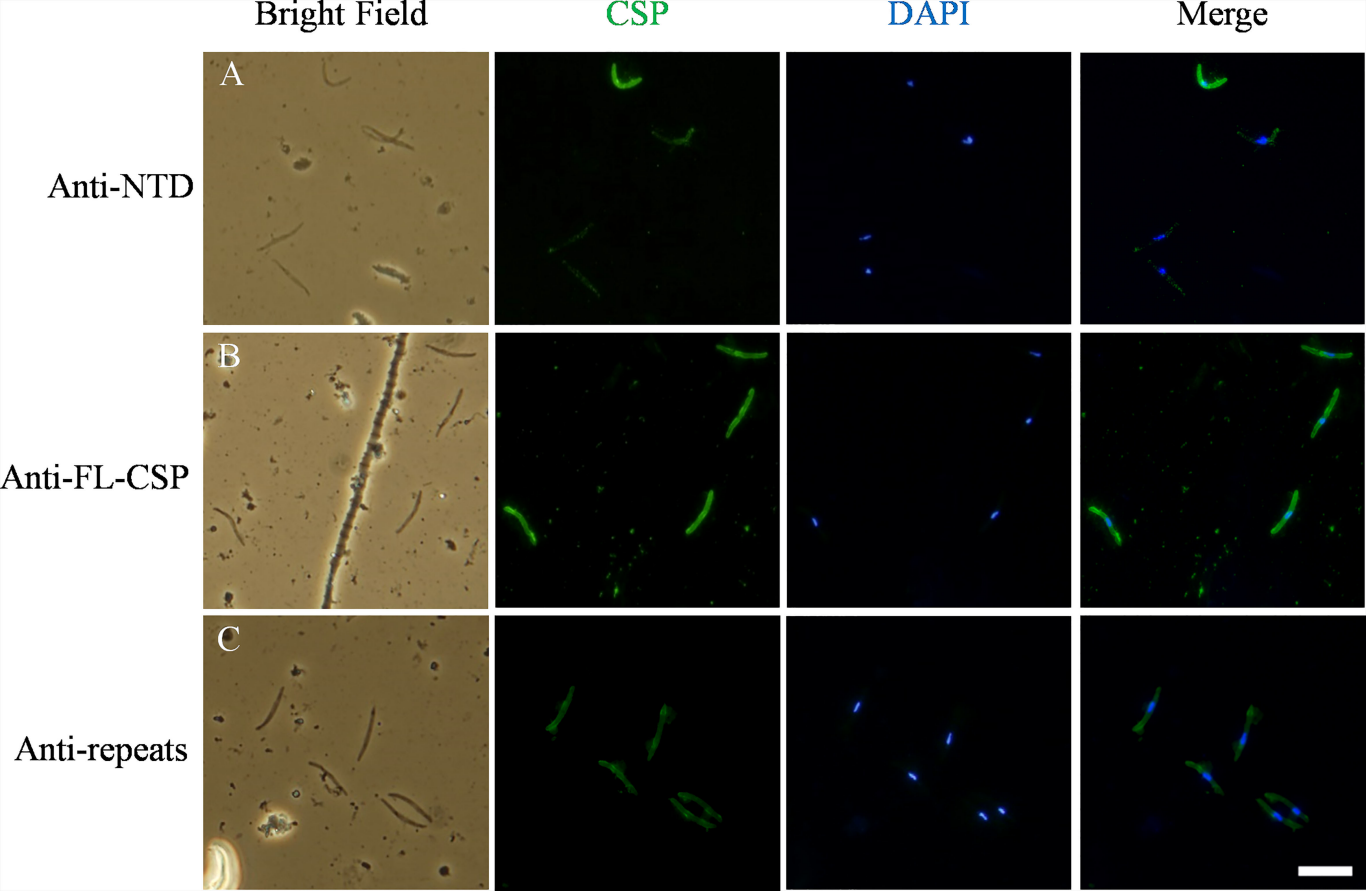
Variable quantities of NTD of CSP are displayed on the surface of SPZs in the in-vitro assays. CSP (green) displayed on the surface of SPZs was stained with antibodies generated against: **(A)** the NTD, **(B)** the full length CSP (FL-CSP) and **(C)** the CSP repeats. Nuclei were stained with by DAPI (blue). Scale bar, 10 µm.

In the experiments described above, we confirmed binding of C4bp to the SPZ surface using IFA. In these experiments, fluorescent signals showing C4bp binding varied from strong to weak. It has been shown previously that CSP becomes proteolytically processed during cell invasion resulting in the removal of its NTD (19). Similar processing was shown to take place also *in-vitro* (19). Therefore, we performed a set of immunofluorescence assays in which antibodies generated against (1) the NTD alone (2), the FL-CSP and (3) CSP central repeats were used to verify the differential presence of the NTD of CSP on SPZs used in our *in-vitro* assays. IFA made with anti-NTD-GST antibodies revealed that not all SPZs stained equally for the presence of NTD of CSP on the parasite surface (**Figure 7**, panel A). However, anti-FL-CSP and anti-CSP-repeat antibodies stained all SPZs to the same extent (**Figure 7**, panel B and C, respectively). This data thus suggests that at least under *in-vitro* experimental conditions not all SPZs display intact FL-CSP. The NTDs of a proportion of CSP molecules were cleaved off as indicated by the weaker signal of anti-NTD-GST antibody staining on some SPZs. This might explain our previous observation that C4bp binding signals on SPZs in IFA were not similar on all SPZs. This is because the quantities of CSP molecules carrying the C4bp binding region, namely the NTD of CSP, vary on the SPZs surface due to the proteolytic cleavage of the NTD that takes place *in-vitro*.

### CSP-C4bp binding motif induces an antibody response in immunized mice

**Figure 8:**
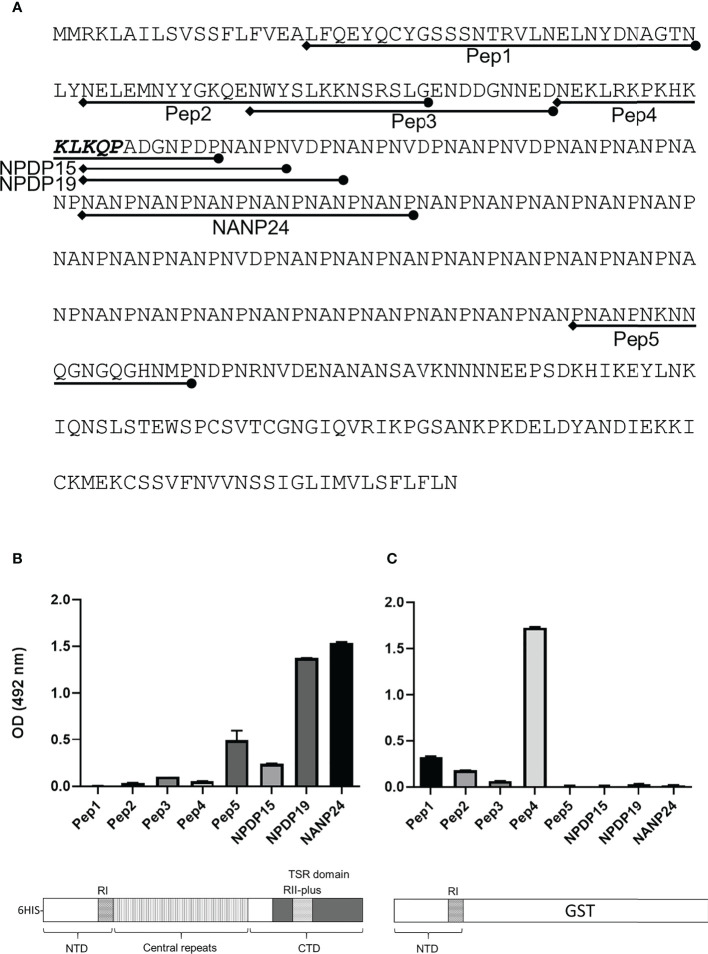
Antibody response against CSP peptides. Mice were immunized with either FL-CSP or NTD-GST. **(A)** Sequences of CSP peptides tested in the ELISA assays delineated on the amino-acid sequence of CSP. **(B)** Antibody levels in a serum pool of 4 mice immunized with FL-CSP against CSP peptides representing the NTD (Pep1, Pep2, Pep3, and Pep4), junction region (NPDP15 and NPDP19), central repeat region (NANP24) and the CTD (Pep5). **(C)** Antibody levels in a serum pool of 4 mice immunized with NTD-GST.

Mice were immunized with FL-CSP to test whether the CSP-C4bp binding motif on the NTD of CSP is a target of an antibody response. A serum pool (diluted 1:1000) from 4 immunized mice was tested in ELISA against biotinylated peptides representing the CSP-C4bp binding peptide (Pep1), the control peptides Pep2 & Pep4 (non-C4bp binding peptides) and Pep3 & Pep5 representing the two other C4bp binding signals from the PepSpot assay (**Figure 8A**). ELISA data showed poor recognition of peptides Pep1-4 with anti-FL-CSP serum pool (**Figure 8B**). On the other hand, Pep5 was better recognized by anti-FL-CSP than Pep1-4 most likely because it contains a single unit (NANP) of the immunodominant central repeat region of CSP. However, the serum pool strongly recognized the NANP24 peptide representing 6 units of the central repeat region of CSP (**Figure 8B**). Antibody titer against the NANP24 peptide was 1:32,000. The NPDP19 peptide (25), representing the junction region between the NTD and the central repeat region was also better recognized by the anti-FL-CSP serum pool (**Figure 8B**) with an antibody titer of 1:32,000. On the other hand, when the central repeat and the CTD of CSP were replaced by the GST protein and used to immunize mice, the NTD-GST protein induced an antibody response against Pep1 (C4bp-CSP binding peptide) (**Figure 8C**) although at a much lower titer (1:2,000). Interestingly, anti-NTD-GST serum pool recognized the control peptide, Pep4 (**Figure 8C**), that contains the highly conserved RI (KLKQP sequence) present in CSP from all species of *Plasmodium*, with a titer (1:32,000) similar to that measured against the 6 repeats peptide (NANP24) of the repeat region when Fl-CSP was used as an immunogen. Altogether, this data suggests that the NTD of CSP became immunogenic only when it was taken out of the context of the full length CSP.

## Discussion

Exposure of the developmental stages of the malaria parasite to human complement within the complex life cycle of the parasite is evident, as is the need to escape immune attack. The invasive extracellular SPZs that are inoculated *via* a mosquito bite into human skin are one of those stages. SPZs take up to 30 minutes to reach the liver from the inoculation site. They are exposed to complement during this journey, yet many of them succeed in infecting hepatocytes. Moreover, individuals living in malaria-endemic areas are only protected from malaria symptoms after repeated infections with the malaria parasite over several years (26). This suggests that antibody responses induced by multiple exposure to infectious loads of SPZs do not easily block infection. One possible explanation for this phenomenon would be the ability of SPZs to escape complement effector functions by inhibiting complement activation on their surfaces. Indeed, other *Plasmodium* species, *P. berghei*, and *P. gallinaceum*, were reported earlier to be resistant to the host complement system, mouse, and chicken, respectively, and that this resistance seemed to be host and stage (oocyst versus salivary gland SPZs) specific (27, 28). These findings suggested that SPZs could have evolved complement evasion mechanisms. Recent studies also demonstrated that other developmental stages of the *P. falciparum* parasite, merozoites and gametes, have surface molecules that can capture host soluble complement regulators, particularly the alternative pathway inhibitor FH, to inhibit complement activities and to avoid cell damage (5–7).

The current study sheds some light on the evasion of the complement system by the SPZ stage of *P. falciparum* parasite. We have shown that SPZs immobilize the complement regulator C4bp on their surface. This was shown by radioligand, ELISA and immunofluorescence assays. The major SPZ surface protein, CSP, was identified as the C4bp-binding partner on the SPZ surface. In PepSpot assay C4bp binding signals were observed on 3 regions on CSP, two on the NTD and one at the junction region between the repeats and the CTD. However, further analyses using biotinylated peptides in ELISA showed signal 1 on the NTD with 4 positive spots on PepSpot membrane to be the potential binding motif. PepSpot positive binding signals with less than 4 positive spots were shown to be false positive binding signals suggesting that PepSpot signals represented by a high number of overlapping peptides (4 peptides in the case) were likely to represent a true binding signal. Interaction between the NTD of CSP and C4bp was also confirmed using a recombinant NTD to demonstrate that peptide binding to C4bp was not an artefact. A soluble complement regulator, FH, was used as a negative control for this assay and showed no significant binding to recombinant NTD. CCP1-2 binding and inhibition assays mapped the NTD-binding site on C4bp to this region, although other C4bp α-chain domains could also been involved but this was not investigated in the current study. However, this finding was consistent with previous reports showing that CCP1-2 domains contain the binding site on C4bp to many pathogens (13). Gliding motility is considered a critical activity for SPZs to access the circulation at the mosquito bite site (22, 29). SPZs gliding motility was reduced in the presence of malaria hyperimmune IgG while C4bp was absent from NHS. Even though the reduction in motility was statistically significant, it was only modest. This modest inhibition could be due to low levels of anti-CSP IgG, required to activate the classical pathway, in the hyperimmune IgG used in the study or other unidentified complement evasion mechanisms present in the SPZ stage. Nevertheless, slowing down the gliding motility of SPZs within the dermis after a mosquito bite could allow for sufficient time for the phagocytic cells to clear them before they reach the circulation. This inhibitory effect was abolished when C4bp-depleted NHS was replenished with C4bp confirming the protecting role of C4bp to the SPZs i.e., controlling complement activation on the parasite surface. The inhibitory effect was only observed when >10 gliding circles were considered in the analyses since SPZs with only 1 or 2 circles were likely to represent some unfit SPZs resulting from the SPZ isolation procedure.

We have also shown that an antibody response against the NTD, containing the C4bp binding motif (Pep1), was undetectable if mice were immunized with FL-CSP suggesting that the repeating identical immunogenic determinants within the central repeat domain can mask immune responses to other CSP domains. However, anti-NTD antibodies became detectable, when the central repeats and the CTD were removed from the antigen used for immunization (NTD-GST). Even though anti-Pep1 antibodies were observed when immunising mice with NTD-GST, the highest observed antibody response was to a 22 amino acids long RI-containing peptide (Pep4). A similar finding has been reported in earlier studies in which a motif upstream RI was described as an immunologically cryptic epitope in mice immunization studies (30), and the same phenomenon has been observed for rabbit immunization (31). A recent study suggested that CSP central repeats could act as a decoy that prevents the adaptive immune system from eliciting humoral responses against other CSP domains i.e., NTD and CTD (32). It was suggested (32) that the primary driver of the immunodominance of the CSP repeats over other domains within CSP is the avidity of the binding between long repeats and BCRs on the surface of antigen-specific B cells (competition in the germinal centres between B cell clones). Here, CSP molecules carrying long repeats may be readily taken up by CSP repeats-specific B cells, allowing these B cells to outcompete CSP NTD- and CSP CTD-specific B cells for T cell help. However, no clear experimental data was given on this hypothesis, the main finding was that reducing the number of repeats in CSP increases the antibody responses to the NTD and CTD domains and that altering the number of B cell precursors specific for different CSP regions in naïve mice did not suppress the immunodominance effect of the CSP repeats (32).

Interestingly, an earlier report showed an association of the antibody response to the NTD of CSP with protection from infection in a cohort of children (33). Moreover, two recent studies (25, 34) have identified a previously unidentified epitope at the junction between the NTD and the central repeat domain. Human monoclonal antibodies targeting this epitope were shown to confer high-level, sterile protection *in vivo* (34). Thus, our current findings together with the growing evidence on the importance of NTD in protective immunity against malaria warrant further investigations to understand whether these newly identified epitopes and the C4bp binding motif on the NTD are essential for CSP function.

Collectively, our results provide insights into complement immune evasion by the SPZ stage of the *P. falciparum* malaria parasite. The evasion mechanism was shown to involve interaction of the NTD of CSP on the surface of the SPZ and the complement regulatory protein C4bp. Preventing this interaction resulted in reduced SPZ motility, a vital activity of the SPZs.

Incorporating the CSP-C4bp binding region into a CSP-based vaccine formulation could induce vaccine-mediated immunity that neutralizes this immune evasion region and increases the vaccine efficacy.

## Materials and methods

### Human serum

NHS was produced from blood collected from 7–10 healthy adults working in the laboratory with written informed consent and used anonymously. The blood was then allowed to clot, and the serum was subsequently harvested, pooled, and stored at −70°C until used. HIS was generated by incubating NHS for 1 h at 56°C.

### Radioligand assay

Salivary glands were dissected from both *P. falciparum*-infected (NF54 strain) and uninfected *Anopheles stephensi* mosquitoes reared in the Radboud University Medical Center insectary (Nijmegen, The Netherlands). *P. falciparum* SPZs were isolated from salivary glands using glass homogenizer in PBS and counted under a phase contrast microscope using a hemocytometer. SPZs were pelleted by centrifugation at 16,000 x g for 5 min at 4°C and resuspended in 1xGVBS buffer (1x VBS, pH 7.2 + 0.1% gelatin). Twenty microliters of SPZ suspensions of 3 different SPZ counts (10^5^, 3x10^5^ and 10^6^) were incubated with 20 μl of ^125^I-C4bp (∼20,000 cpm/sample) for 30 min at 37°C with agitation. After incubation, the samples were centrifuged through 250 μl 20% sucrose in GVB at 10,000 × g to separate free protein from SPZ-bound proteins. After separation, radioactivities in the pellet and in the supernatant were measured in a gammacounter. The ratios of bound to total radioactivity were calculated. In these assays *Fusobacterium necrophorum* (∼4 × 10^7^ cells/assay) was used as the positive control. Salivary glands’ insoluble fractions (equivalent to those that yielded 10^6^ SPZs) from uninfected mosquitoes were used as the negative control.

### Whole-SPZ ELISA

MaxiSorp 96-well microtiter plates (Nunc) were coated with 10^4^ SPZs/well or salivary glands’ insoluble fraction (equivalent to those that yielded 10^4^ SPZs) from uninfected mosquitoes. After overnight incubation at 4°C, wells were washed thrice with PBS and blocked with 1% fatty acid free BSA (Sigma) in PBS for 1h at room temperature (RT). Following blocking, 50% HIS or VBS^++^ was applied in triplicate to the wells for 1h at RT. After three washes with PBS, wells were incubated with 1:500 dilution of sheep polyclonal anti-human C4bp (PC026, The Binding Site) in 1% fatty acid free BSA in PBS for 1h at RT, then washed thrice with PBS. Next, wells were incubated with 1:2000 donkey anti-sheep IgG/HRP (713-035-147, Jackson ImmunoResearch) for 1h at RT. Finally, wells were washed thrice with PBS and C4bp binding was detected colorimetrically by using tetramethylbenzidine substrate (Thermo Fisher Scientific) and reading at 450 nm using the HIDEX Sense plate reader.

### Immunofluorescence staining

SPZs that were cryopreserved according to a previously described protocol (35) were thawed at RT and washed three times with VBS^++^ (1x VBS, pH 7.3, 0.15mM CaCl_2_, 0.5mM MgCl_2_). Washed SPZs were then incubated with 50% HIS in VBS^++^ or with VBS^++^ (negative control) for 30 min at 37°C. Following incubation, SPZs were washed three times with VBS^++^ and resuspended in DMEM containing 3% BSA. Next, SPZs (5x10^4^ SPZs per well) were added to Lab-Tek wells (Nalgene, Nunc) previously coated with the anti-CSP repeats monoclonal antibody (3SP2) (36) and allowed to sediment onto the bottom of the wells for 1h at 37°C. The supernatant was then carefully removed and SPZs were fixed with 4% paraformaldehyde (PFA) in PBS and washed. Nonspecific binding was blocked with 1% BSA in PBS. SPZ were then incubated with 1:500 dilution of sheep polyclonal anti-human C4bp (PC026, The Binding Site) or in blocking solution without primary antibodies (negative control) for 1h at 37°C. After three washes with PBS, SPZs were stained for 1h at RT with a combination of 0.5 μM DAPI (4′,6-diamidino-2-phenylindole) and 1:400 dilution of donkey Alexa Fluor 488 anti-sheep IgG (A-11015, Invitrogen). Following SPZ staining, Lab Tek wells were washed three times with PBS, the chamber was removed, and a coverslip was mounted onto the slide using Mowiol-based antifading medium. Slides were kept at 4°C degrees prior to examination with the Olympus BX51 fluorescence microscope. Images were captured using the Olympus DP70 camera with the help of DP controller software.

For the specific immunostaining of the different CSP regions, freshly isolated *P. falciparum* SPZs were spun onto a slide using the cytospin (Shandon Cytospin 2), fixed with cold methanol and kept at -20°C degrees. On the day of the experiment, the slides were removed from the freezer and SPZs were blocked with 1% BSA in PBS for 30 min at RT. Next, slides were washed once with PBS for 5 min and incubated at 37°C for 1h with either 1:1000 dilution of mouse polyclonal anti-full length-CSP (FL-CSP), 1:100 dilution of mouse polyclonal anti-NTD-GST, 1:200 dilution of anti-CSP repeats monoclonal antibody (3SP2) or with blocking solution (negative control). Following incubation, slides were washed three times in PBS/Tween-20 0.05% (PBS-T) (5 min per wash) and SPZs were stained with 1:500 dilution of goat Alexa Fluor 488 anti-mouse IgG (A-11001, Invitrogen) and 1 μM DAPI. Finally, the slides were washed again in PBS-T, mounted, and analyzed as described above.

### 3D7 CSP sequence

The DNA sequence of the *csp* gene (Gene ID: 814364) of the *P. falciparum* 3D7 strain was retrieved from the NCBI’s Gene database. Translated protein sequence was used for peptide synthesis and the DNA sequence was used to synthesize PCR primers to amplify sequences from 3D7 genomic DNA for protein expression.

### Peptide scanning using membrane-bound peptide array

Peptides spanning FL-CSP sequence, residues 1–397, were synthesized and covalently attached *via* carboxyl termini to polyethylene glycol-derivatized cellulose membranes (AIMS Scientific Products) using the peptide scanning instrument AutoSpot Robot ASP222 (Abimed Analysen-Technik). Peptides were synthesized as 92 16-amino acid-long peptide spots (PepSpot) with a three amino acid shift from one spot to the next (13 amino acid overlap). After blocking with 4% skim milk for 1h at RT, the PepSpot membrane was incubated with radiolabeled C4bp (1 × 10^6^ cpm) in 10 ml PBS-T for 4 h at RT. After extensive washing with PBS-T, binding was detected by exposure on a phosphoimager plate and Fujifilm BAS 2500 instrument (Fuji Photo Film).

### Biotinylated peptides

Three CSP peptides (Pep1, Pep3 and Pep5, **Figure 2D**) representing the C4bp binding signals (3 signals of 4, 3 and 2 overlapping peptide spots, **Figure 2B**) on the PepSpot membrane were synthesized by Proteogenix (Schiltigheim, France). Two more peptides (Pep2 and Pep4, **Figure 2D**) were synthesized as controls. The quality of the soluble peptides was assessed with matrix‐assisted laser desorption/ionization time of flight (MALDI‐TOF) mass spectrometry. The peptides were biotinylated on their C‐termini. The previously published peptides NPDP15 and NPDP19 (on the junctional region between the NTD and the central repeat regions of CSP) and NANP24 (representing 6 central repeats) (25) were synthesized similarly. Peptides were dissolved in 75% DMSO in PBS.

### ELISA-based C4bp-CSP peptide binding assay

MaxiSorp 96-well microtiter plates (Nalgene, Nunc) were coated with 100µl/well, 5 µg/ml C4bp (Complementech) diluted in PBS. After an overnight incubation at 4°C, C4bp solution was decanted, and the wells were washed once with PBS-T. Wells were then blocked with 1% casein in 200µl PBS-T for 1h at RT. Blocking buffer was decanted and wells were washed thrice with PBS-T. Biotinylated peptides were diluted to the desired concentration 8-0.125 µM (2-fold serial dilutions) and 100µl was added to each well. After a 3h incubation at RT, wells were washed 5 times with PBS-T and incubated for 1h with 100 µl of avidin-HRP (Vector Laboratories), 2.5 µg/ml, to detect bound peptides. Wells were washed 5 times with PBS-T, and 100 µl of the substrate o-phenylenediamine dihydrochloride (OPD, 34006, Thermo Scientific) were added to each well. The color development was observed, and the reaction was stopped with 100 µl H_2_SO_4_ and read at 492 nm using the HIDEX Sense plate reader.

### Protein expression

The DNA sequence of the NTD sequence of CSP, residues 17-104, was amplified by PCR using sequence specific primers (NTD-GST-F: TGGGATCCAGGAGGCCTTATTCCAGGAA and NTD-GST-R: CACTCGAGCTA TGGATCTACATTTGGGTTGGCAT) with BamHI and XhoI restriction sites, respectively. Amplified sequence was cloned into BamHI and XhoI sites of the pGEX-5X-1 plasmid. NTD-GST protein expressed in *Escherichia coli* strain BL21(DE3) was purified under native condition using affinity chromatography on Glutathione Sepharose (GE healthcare). The same sequence was also amplified by PCR using sequence specific primers (NTD-HIS-F: TGCATATGGAGGCCTTATTCCAGGAA and NTD-HIS-R: CAGGATCC CTATGGATCTACATTTGGGTTGGCA) and cloned into the Nde I and BamH I restriction sites of the pET15b (Novagen) vector to express NTD fused to 6-histidine residues, NTD-H. Expressed protein was purified under native condition on a HisGraviTrap column (GE healthcare). Clone pDS56 –32/RBSІІ – CS27IV C - 6X His (MRA-272, MR4, ATCC® Manassas Virginia) expressing most of the N- and C-terminal regions and a full representation of the repeat domain i.e. (27-123(NANPNVDP)_3_(NANP)_21_300-411) from the T4 *P. falciparum* strain was used to express the full length CSP (FL-CSP). FL-CSP was purified under native condition on a HisGraviTrap column (GE healthcare). Purity of the recombinant proteins was verified by SDS-PAGE.

Recombinant CCP1-2 of the C4bp α-chain was expressed and purified as described previously (37), with some adaptations. Briefly, the pET26 plasmid (Novagen) carrying the DNA coding sequence for CCP1-2 was transformed into the SHuffle^®^ competent *E. coli* and the protein expression was induced with IPTG. The protein was expressed as inclusion bodies that were isolated by cell lysis. The inclusion bodies were resuspended in 6 M Guanidium-HCl, 20 mM Tris-HCl at pH 8.0 in the presence of 10 mM reduced glutathione and the recombinant protein was purified using a HisTrapTM FF column (Cytiva). Following purification, the protein was diluted to 0.08 mg/mL in refolding buffer at pH 8.5 (10.56 mM NaCl, 55 mM Tris-HCl, 2.2 mM CaCl2, 2.2 mM MgCl2, 0.055% PEG4000, 0.55 M L-arginine, 0.1 mM oxidized glutathione, and 1 mM reduced glutathione) and incubated overnight at 4°C with shaking. The next day, the refolding solution was incubated with iodoacetamide (5 mM) for 30 min and dialyzed against 50 mM Tris-HCl at pH 8.5. Finally, the correctly folded protein was purified using a Resource™ Q column (Cytiva). Purity of the recombinant proteins was verified by SDS-PAGE and the functionality was monitored by studying the binding of purified C4b (Comptech) to the recombinant protein in an ELISA-based assay.

### ELISA-based C4bp-NTD-H binding assay

Purified C4bp and FH were diluted to 2 µg/ml with PBS, and 100 µl/well were used to coat MaxiSorp 96-well microtiter plates (Nalgene, Nunc). After an overnight incubation at 4°C, wells were washed once with PBS-T and blocked with 1% casein, 200 µl/well for 1h. Wells were then decanted and washed once with PBS-T. Six concentrations (1000-31nM) of 2-fold serial dilutions of NTD-H were prepared in VBS^++^ buffer and added to the respective wells. After a 2h incubation at RT, wells were washed 5 times with PBS-T. Anti- NTD-GST antibodies in 1:1000 dilution in PBS-T were added to wells (100 µl/well) and incubated for 1h at RT. After 5 washes with PBS-T, 100 µl of 1:3000 dilution of goat anti-mouse IgG/HRP (P0447, Dako) were added to wells and incubated at RT for 1h. Wells were washed 5 times with PBS-T and binding of the antibodies to bound NTD-H were detected colorimetrically by using the OPD substrate and reading at 492 nm using the Labsystems IEMS reader.

### Co-immunoprecipitation

The interaction between CSP derived from SPZs with human C4bp was analyzed by a standard co-immunoprecipitation assay. Cryopreserved SPZs (5x10^5^) were thawed at RT, washed three times in VBS^++^ and incubated with either 50% HIS in VBS^++^ or VBS^++^ (negative control) for 30 min at 37°C. Following incubation, SPZs were washed three times in PBS-T supplemented with protease inhibitor tablets, EDTA Free (88266, Thermo Scientific). Next, SPZ protein extracts were obtained by incubation with lysis buffer (0.5% Triton X100, 50 mM Tris-HCl, 75 mM NaCl, pH 8) supplemented with protease inhibitors at 4°C with shaking for 30 min, followed by centrifugation at 16,000g at 4°C for 30 min. The pellets were discarded and a 20 μl samples from each condition (inputs) were prepared for Western blot analysis. The lysates were then incubated with anti-C4bp (PC026, The Binding Site) coated Dynabeads™ Protein G (10004D, Invitrogen) with shaking for 1h at 4°C. Later, beads were separated from the supernatants (SN) and 20 μl samples from each condition (SN) were analyzed by Western blotting. Following this step, beads were washed three times with lysis buffer and the co-immunoprecipitates (co-IP) were analyzed by Western blot analysis. co-IP, inputs and SN samples were prepared with x1 Bolt™ LDS sample buffer (B0008, Thermo Scientific) containing x1 Bolt™ sample reducing agent (B0009, Thermo Scientific), heated for 5 min at 90°C and loaded on a gradient electrophoresis gel (NW04120BOX, Invitrogen). Separated proteins were transferred to a nitrocellulose membrane, which was blocked by PBS-T containing 3% milk for 1h at RT. Next, the membrane was cut horizontally into two pieces under the 75kDa protein ladder mark and probed with 1:1000 dilution of sheep polyclonal anti-human C4bp (PC026, The Binding Site) (upper membrane piece) or 1:1000 dilution of mouse monoclonal anti-CSP repeat (3SP2) (lower membrane piece) overnight at 4°C. The next day, membranes were washed with PBS-T and primary antibody binding was detected by incubation with 1:20,000 dilution of donkey anti-sheep IgG/HRP (713-035-147, Jackson ImmunoResearch) (upper membrane piece) and 1:20,000 dilution of goat anti-mouse IgG/HRP (P0447, Dako) (lower membrane piece) for 1h at RT. The blot was then developed by ECL Western blot analysis system-based detection followed by exposure to Super RX film (47410-19236, Fujifilm).

### Preparation of C4bp-depleted NHS

To prepare C4bp-depleted NHS, 20% fresh NHS pool in VBS was passed thrice at 4°C through a HiTrap™ NHS-activated HP column coupled to M22-N peptide (38). After depletion, C4bp-depleted NHS was supplemented with calcium and magnesium (final concentration 0.15 mM and 0.5 mM, respectively). C4bp depletion was confirmed with Western blot analysis with sheep polyclonal anti-C4bp (PC026, The Binding Site) and donkey anti-sheep IgG/HRP (713-035-147, Jackson ImmunoResearch). To rule out the possibility of reduced complement activity in the C4bp-depleted NHS pool due to C4bp depletion treatment; the classical/mannose/alternative pathways in the C4bp-depleted NHS were evaluated for their complement activity using Wieslab^®^ complement system screen (SVAR) according to the manufacturer’s instructions.

### Gliding motility assay

8-chamber glass Lab-Tek wells (Nalgene, Nunc) were coated overnight at RT with 5 μg/ml anti-CSP repeats mAb (3SP2) in PBS. The next day, freshly dissected SPZs were incubated with 10% (v/v) NHS/HIS or C4bp-depleted NHS/HIS in VBS^++^ (final volume of 100 µl per condition including 25 µl 3% BSA in DMEM) for 10 min at 37°C. In addition to human serum, SPZs were also incubated with different combinations of 1:10 pooled human IgGs from 10 women from a malaria endemic area who were *P. falciparum*-positive at the time of delivery (39), purified with Melon Gel (Thermo Fisher Scientific); and/or 25 µg/ml of commercially purified C4bp (CompTech), when indicated. Next, treated SPZs were diluted in DMEM 3% BSA and added to the 3SP2 coated Lab-Tek wells (~30 000 SPZs/well) and allowed to glide for 1h at 37°C. Following incubation, the supernatant was removed, and wells were fixed with 4% PFA in PBS, washed and blocked with 1% BSA in PBS. SPZs were then incubated with 2.5 µg/ml of anti-CSP-FITC mAb (3SP2) in blocking solution for 1h at 37°C. After three washes, the chamber was removed, and a coverslip was mounted onto the slide using Mowiol-based antifading medium. Slides were kept at 4°C degrees prior to examination with the Olympus BX51 fluorescence microscope. For each well, 100 SPZs were counted and for those SPZs associated with trails, the number of circles for each trail was counted.

### Production of polyclonal antibodies

Female C57BL/6 Mice (6–9 weeks of age) were immunized in day 0 with 50 μg FL-CSP or NTD-GST emulsified in complete Freund’s adjuvant, followed by a booster dose on day 14 of proteins emulsified in incomplete Freund’s adjuvant. Mice were bled on day 28. Recovered sera were used for immunostaining and ELISA assays. Animal procedures were approved by the Board for Animal Research (ELLA), Southern Finnish State Administrative Agency (ESAVI/11326/2020)

### Measuring serum antibody response in peptide ELISA

Streptavidin 96-well microtiter plates (Invitrogen) were coated with 0.5 μg/ml biotinylated peptides in PBS. After overnight incubation at 4°C the plates were washed 3 times with PBS-T. Mouse serum samples were diluted 1:1000 with 1% BSA/PBS-T and 100 µl were added to the respective wells and plates were incubated 2h at 4°C. Serial dilutions of serum samples were also made to measure antibody titer against selected peptides. Wells were washed 5 times with PBS-T and 100 µl of 1:2000 diluted goat anti-mouse IgG/HRP (PerkinElmer) were added to each well. After 1 h incubation at 4°C plate wells were washed 5 times with PBS-T and 100µl of OPD were added to each well. Color development was observed, the reaction was stopped with 100 µl H_2_SO_4_ and plates were read at 492 nm using the HIDEX Sense plate reader. The ELISA’s cut-off level was set to twice the mean absorbance obtained from the blank wells.

### Statistical analysis

Data are presented as means and standard deviations (SD). Shapiro-Wilk test for normality was performed and for data sets returning normal distribution, *t* test was used when comparing two groups and one-way analysis of variance (ANOVA) followed by *post-hoc* Tukey’s multiple pairwise comparisons was used when comparing more than two groups. For non-normal distributions, The Mann-Whitney test (for two groups) and Kruskal-Wallis test (for more than two groups) followed by *post-hoc* Dunn’s multiple comparisons test were used to carry out statistical analysis of the data. A probability value of P < 0.05 was indicative of statistical significance in all tests. All calculations were performed using the GraphPad 9.1.0 software package. All experiments were performed in triplicate.

## Data availability statement

The raw data supporting the conclusions of this article will be made available by the authors, without undue reservation.

## Ethics statement

The animal study was reviewed and approved by The Board for Animal Research (ELLA), Southern Finnish State Administrative Agency.

## Author contributions

Conceptualization, AK and SM. investigation, AK, MR, MB, MK, and TP. formal analysis, AK and MR. resources, AK, MK, TP, TF, G-JG, TB, PP, OT, RS, AL and SM. writing–original draft, AK and MR. writing–review & editing: all authors. supervision, AK and SM. funding acquisition, AK and SM. All authors contributed to the article and approved the submitted version.

## Funding

Research funding to SM and AK was provided by the Jane and Aatos Erkko foundation (4706167), the Academy of Finland (1323237), the Sigrid Jusélius Foundation (4705080), and the Helsinki University Hospital Funds (TYH2019311). MR was supported by funds of the EU MSCA project CORVOS 860044.

## Acknowledgments

We thank Anna Blom for donating the C4bpα CCP1-2 expression plasmid (pET26-CCP1-2). The following reagent was obtained through BEI Resources, NIAID, NIH: Plasmid pDS56-32/RBSII-CS27IVC-6XHis, MRA-272, contributed by Photini Sinnis.

## Conflict of interest

The authors declare that the research was conducted in the absence of any commercial or financial relationships that could be construed as a potential conflict of interest.

## Publisher’s note

All claims expressed in this article are solely those of the authors and do not necessarily represent those of their affiliated organizations, or those of the publisher, the editors and the reviewers. Any product that may be evaluated in this article, or claim that may be made by its manufacturer, is not guaranteed or endorsed by the publisher.
